# A comparative evaluation of pharmacy services in single and no pharmacy towns

**DOI:** 10.1186/1743-8462-3-8

**Published:** 2006-06-26

**Authors:** V Bruce Sunderland, Suzanne D Burrows, Andrew W Joyce

**Affiliations:** 1 School of Pharmacy, Curtin University of Technology, GPO Box U1987, Perth, WA 6845, Australia; 2 WA Centre for Health Promotion Research, Curtin University of Technology, Australia

## Abstract

**Background:**

Recent attention has focused on access of communities to pharmacy services in rural areas. To increase access to pharmacy services in rural Western Australia some doctors have been granted a licence to dispense medication on the rationale that a pharmacy would not be economically viable in that community. However, there have been no studies conducted on whether a doctor dispensing service adequately provides a pharmacy service with respect to access and quality.

**Method:**

Residents of seven single pharmacy towns and seven non-pharmacy rural towns were surveyed to evaluate pharmacy services delivered by a pharmacist and doctor. The towns were chosen to match closely on key demographic features, with an average population of 1,246 and 1,263 respectively. A random sample of 150 households from each town was sent the questionnaire on pharmacy services (1050 in each group). Data was also collected from the Health Insurance Commission (HIC) on dispensing locations for the residents of the two groups of towns.

**Results:**

There was a significant difference in access to pharmacy services with 82.4% of participants from pharmacy towns accessing medications within their town compared to 51.3% of non-pharmacy town participants. The HIC data supported these trends with pharmacy town residents having relatively higher prescription rates within their town compared to non-pharmacy town residents where they were more likely to access prescriptions out of their town.

**Conclusion:**

Pharmacy town participants were more satisfied with access to health and pharmacy services within their town. Continuation of the doctor dispensing policy requires a greater consideration of the pharmacy needs of rural residents.

## Background

In rural areas, pharmacists often take on an extended health care role, offering cognitive or counselling services as well as standard dispensing services, and organise other health professionals to work in their pharmacy or local area [1-3]. However, not all residents of Western Australia are receiving the same level of pharmacy care. In Western Australia in 2000 there were 359 urban pharmacies and 110 rural pharmacies, with each urban pharmacy serving approximately 3 923 people and each rural pharmacy serving approximately 4 572 people [4]. Kaiser [5] states that the present spatial location of pharmacies in Western Australia is less than optimal, in terms of accessibility for the entire population. He concluded that travel distances could be reduced if the current number of pharmacies were better arranged [5].

One alternative to the problem of accessing some common pharmacy medicines for rural people may be to introduce telepharmacy in select locations [6]. This may involve using computerised video link ups, the Internet and telephones [6-8] and automatic dispensing machines to provide common dosages of certain frequently used medicines under the supervision of an authorised health care provider, such as a doctor or nurse [7,9]. Mobile pharmacies, whereby a pharmacist travels with supplies and rotates among several small towns that do not have a pharmacy, may assist in improving pharmacy access for residents in such towns, providing licensing regulations are taken into account [10,11]. Such initiatives may be helpful in reducing the need for rural residents to travel to other towns for their medicines or, alternatively, have to wait a few days for a prescription to be dispensed [12].

Another potential solution for increasing access to pharmacy services is general practitioners performing a dispensing role. The granting of doctor dispensing licences was an initiative designed to improve access to pharmaceutical care in rural areas that could not support a pharmacy practice [13]. However, if licenses were granted to areas that could reasonably support pharmacies then this policy could lead to the loss of business for smaller pharmacies and deprive the local community of access to pharmaceutical services [13]. There is no published data in Western Australia to indicate on what demographic basis doctor dispensing licences have been granted. Further, there is no research to indicate whether residents of towns receiving pharmaceutical care through the doctor are receiving the same level of service as residents with access to a pharmacy in their town. Nor is there any data as to the level of patronage of doctor dispensing services relative to pharmacy services in rural areas.

A pilot study was conducted in 2003 to examine the impact of pharmacy services in a small rural community by comparing two towns that had access to a pharmacy service versus two towns that did not have access to a pharmacy service and used a doctor dispensing service [14]. The study also included items on nursing post use. Staffed by a Remote Area Nurse, these centres provide accident and emergency care and have recently expanded their role to that of primary health care provider [15], providing services such as diagnosing and treating minor illnesses, performing minor procedures and ordering basic medical tests [16,17]. The major finding of the pilot study was that people in non-pharmacy towns had poorer access to prescription and non-prescription medicines [14]. The pilot study was limited by discrepancies in town population sizes and this study surveyed a larger sample with towns matched closely on key demographic factors. The objectives of the study were:

1. To determine the pattern of patronage of pharmacy and doctor services in small rural towns.

2. To determine access and availability of prescription and non-prescription medicines in rural communities.

3. To provide data on how minor ailments are managed in towns with and without pharmacies.

This study was approved by the Human Research Ethics Committee, Curtin University of Technology (HR 221/2002).

## Method

### Participants

#### Selected towns

Fourteen towns were selected for the study, seven towns with a single pharmacy service and seven towns with a doctor dispensing service. The authors could access no public records of where doctor dispensing licenses have been granted. Through consulting with health services in rural towns a list of ten towns that had a doctor licensed to dispense medications were identified. From this list seven towns were selected for the study based on being able to locate a corresponding single pharmacy town that matched closely on key demographic variables. Averages of these demographic variables for the pharmacy towns and non-pharmacy towns as taken from Australian Burueau Statistics (ABS) [18] are displayed in Table 1.

**Table 1:** Summary statistics for towns with a pharmacy service and towns with a doctor dispensing service.

	**Pharmacy Towns**	**Non-pharmacy towns**
Shire Population	1246.1	1263.9
Indigenous (%)	5.0	3.3
Birthplace Australia (%)	84.8	86.4
Home Language English (%)	94.0	94.6
		
Age (median)	38.0	34.3
Age (%)		
0–14	23.8	26.1
15–24	8.6	9.6
25–44	28.8	31.3
45–64	25.0	23.4
65+	13.8	9.5
		
Gender		
Female	48.9	45.8
Male	51.1	54.2
		
Income (median)	300 – 400	300 – 400
		
Education (%)		
Postgrad	1.1	1.4
Undergrad	5.2	5.8
Diploma	17.0	18.9
No post school qual	76.7	73.9
		
Household (average size)	2.5	2.6
		
Marital status (%)		
Married	59.4	60.4
Separated	3.6	2.7
Divorced	6.9	5.6
Widowed	6.6	4.4
Single	23.4	26.9
		
Distance from Perth (kms)	218.1	307.0

Australian Bureau of Statistics 2001

These towns have very similar demographic profiles with respect to home language, education, income, and marital status profile. There was a difference in age profile with more older people in the pharmacy town group and gender with more males in the non-pharmacy towns. The other difference was that the pharmacy towns selected are closer in average distance to Perth.

### Materials

An 11 page questionnaire was designed to be completed by one member of the household on behalf of all people living in the household. It was comprised of three sections. Section one asked respondents to supply demographic data on themselves and if applicable their partner. The partner could complete their own details in the sections provided if that was their preference. The items were age, gender, ethnicity, education level, employment status, length of residence in the town, whether any children resided with them, the ages of these children and whether they were being treated for any illnesses.

Section 2 covered access to primary health services. It was written at the top of the section: "'you' refers to you and your family where applicable." The items asked respondents how often they obtained non-prescription and prescription medicines, where prescriptions were collected from, the distance and time travelled in order to collect medicines, how much was spent on prescriptions in an average month, how often a doctor was visited, the time and distance travelled to reach this doctor (or specialist doctor), how often a community nursing post was visited, the time and distance travelled in order to reach this nursing post, how much was spent on medicines from nursing posts, whether the internet or mail order was ever used to purchase medicines and, if not, would the respondent ever consider using these services in the future. There were also items on where people accessed health and product information with choices provided of pharmacy, doctor, nursing post, local shop, other and unsure/not applicable.

Section 3 of the questionnaire required participants to provide an indication of how strongly they agreed or disagreed with a series of ten statements. The statements included the following – there is too long a delay from when I need a medication to when it is available to me, I have to travel too far to get prescriptions filled, the health services in my town are adequate for my needs and I think my town needs more access to pharmacy services. Table 6 provides the full list of questions and results. The response format used was a five-point Likert scale (1 = strongly agree, 2 = agree, 3 = unsure, 4 = disagree, 5 = strongly disagree). The last two items of the questionnaire provided opportunity for participants to respond to what they saw as being the main benefits of having a local pharmacy and, conversely, what they saw as being the main disadvantages of not having a local pharmacy.

Satisfaction ratings were used as they provide opportunities for clients to evaluate the level of care they receive and they have been shown to predict utilisation of health services and compliance with treatment recommendations [19]. The items were based on Hepler and Strand's [20] definition of pharmaceutical care by concentrating on ability to access medications when needed and information provided about those medications. There were some items that related to health information provision to reflect the broader role of community pharmacists [21]. The wording of these questions was decided upon by a group of pharmacists to reflect what they considered important aspects of pharmacy service and tested on a group of health consumers prior to the commencement of the pilot study to ensure its readability and ease of completion. The questionnaire took approximately ten minutes to complete.

### Procedure

Questionnaires were mailed to a random sample of households for self-administration. Along with the questionnaire, these households were provided with an information letter outlining the project and a consent form. Also attached was a reply-paid envelope. The first mail out date was the 3^rd ^September 2004. The second mail out for non-respondents was on the 2^nd ^November 2004 and included the questionnaire, consent form, and letter explaining the study and why they had received the material twice.

Based on the pilot study 55% of the respondents from a town with a dispensing doctor accessed medications within their town and 90% of respondents from a single pharmacy town accessed medications within their town. Based on these proportions for a significance level of .05 and 90% power the sample size required for each group was only 33. Thus a more conservative strategy was used for selecting sample size. The population size for the pharmacy towns was 8723 and for the non-pharmacy towns 8847 which for the total combined population was an average of 8785. For a population of 8785, a sample size of 350 (50 from each town), and conservatively assuming the percentage of people accessing medications in their own town would be 50%, the 95% confidence interval would be ± 5.1%. The response rate was approximately one third in the pilot study so to achieve a sample size of 50 from each town required sending the questionnaire to 150 households. A random number generator was used to select the 150 households from the shire electoral rolls for each town. If two members from the same family and address were selected the next closest person on the roll list not from that family was selected. Thus for each town a random sample of 150 participants was produced.

In an effort to boost the return rate and to give people prior notice of the study, the project was advertised in local newspapers at the end of August and the start of September. In addition, data reports were obtained from the Health Insurance Commission (HIC). These reports provided information relating to where people in each of the 14 towns, based on residential postcodes, had medicines dispensed for the period of January 1^st ^2003 to December 31^st ^2003.

### Analyses

Descriptive and inferential statistics were conducted using SPSS (Version 11). In the demographic section it was found that the pharmacy towns and non-pharmacy towns were matched very closely on health and demographic variables. The two differences that did emerge were that there were more older people in pharmacy towns relative to non-pharmacy towns and the average distance of the non-pharmacy towns was further from Perth. To ascertain whether these factors influenced the results a series of logistic regression analyses were run. The outcomes chosen were satisfaction with health services in their area and satisfaction with access to pharmacy services. These were chosen as proxy health and treatment outcomes as satisfaction with services has been related to increased compliance with taking medications and continuity of care [22]. A logistic regression model was used to test whether the type of town predicted satisfaction after controlling for age and distance style items. Logistic regression was selected as the most appropriate model as it has less restrictions on variable distributions [23].

For both satisfaction with health services and pharmacy services the categories of strongly agree and agree were collapsed and the categories of strongly disagree and disagree were collapsed into the other category. Those who entered unsure were not entered in the analyses. Age of respondent and partner, location of nearest pharmacy services, location of nearest doctor services, and location of access to emergency medications were entered into the first block of the logistic regression. Towntype, pharmacy town or non-pharmacy town, was entered on the second block to ascertain whether inclusion of towntype contributed to prediction of satisfaction with health services after controlling for the demographic and distance variables. The results of these analyses are presented in the 'Predicting Satisfaction with Services' section of the results.

## Results

### Questionnaire participants

The response rate was 37.9%, pharmacy towns (37.6%) and non-pharmacy towns (39.8%). Overall there were 819 participants and 653 of these participants had partners (79.7%). Of these participants, 513 were female and 300 male. There was no difference in gender ratios, ethnicity (99% Caucasian), education level, and occupation status between the two groups. There was a significant difference in age proportions, χ^2 ^(7, *n *= 803) = 15.77, *p *< .05, which is depicted in Table 2.

**Table 2:** Age frequencies of primary respondents to the questionnaire.

		Pharmacy Town	Non-pharmacy town	Total
18–25	Count	3	4	7
		.8%	1.0%	.9%
26–35	Count	37	38	75
		9.4%	9.2%	9.3%
36–45	Count	72	91	163
		18.4%	22.1%	20.3%
46–55	Count	75	97	172
		19.1%	23.6%	21.4%
56–65	Count	82	95	177
		20.9%	23.1%	22.0%
66–75	Count	66	57	123
		16.8%	13.9%	15.3%
76–85	Count	42	23	65
		10.7%	5.6%	8.1%
86 +	Count	15	6	21
		3.8%	1.5%	2.6%

There were more participants from pharmacy towns in the oldest category and more middle aged respondents from the non-pharmacy towns. The mean number of illnesses for the pharmacy towns was 1.5 and for the non-pharmacy towns it was 1.3. A *t *test revealed there was no significant difference between the groups on number of illnesses, *t *(816) = 1.32, *p >*.05.

### Access to primary health services

There were no differences between the town types in frequency of medicine use with over 70% of participants obtaining both prescription and non-prescription medications either monthly or a few times a year. Table 3 displays the location of where people obtained their medicines.

**Table 3:** Location of medicines obtained for pharmacy towns and non-pharmacy towns.

		Pharmacy Town	Non-pharmacy town	Total
In your town	Count	324	179	503
		82.4%	51.3%	67.8%
In a nearby town	Count	31	38	69
		7.9%	10.9%	9.3%
In a town more than 40 kms away	Count	30	110	140
		7.6%	31.5%	18.9%
In the Perth area	Count	8	20	28
		2.0%	5.7%	3.8%
Other	Count	0	2	2
		.0%	.6%	.3%

Residents from non-pharmacy towns were significantly more likely to access medications in a town 40 kms away, χ^2 ^(4, *n *= 742) = 93.09, *p *< .001. Over 90% of residents from pharmacy towns access medications within 40 kms of their home compared to just over 60% of non-pharmacy town participants. Participants who needed to travel from pharmacy towns travelled an average of 137.1 km compared to 194.7 km for non-pharmacy town residents, *t *(272) = 3.10, *p *< .05. Correspondingly the time difference was 46 minutes longer for the non-pharmacy town residents, *t *(276) = 3.22, *p *< .05.

Table 4 displays which health service was used for emergency and minor ailment treatments by pharmacy town and non-pharmacy town participants.

**Table 4:** The most common health service that participants would use for emergency and minor ailment treatments for pharmacy towns and non-pharmacy towns.

		Pharmacy Town	Non-pharmacy town	Total
Pharmacy	Count	208	71	279
		53.9%	21.0%	38.5%
Doctor	Count	95	120	215
		24.6%	35.5%	29.7%
Nursing Post	Count	13	25	38
		3.4%	7.4%	5.2%
Local shop	Count	7	44	51
		1.8%	13.0%	7.0%
Other	Count	3	15	18
		.8%	4.4%	2.5%
Unsure/not applicable	Count	48	50	98
		12.4%	14.8%	13.5%
Hospital	Count	12	13	25
		3.1%	3.8%	3.5%

The majority of participants from pharmacy towns would access emergency and minor ailment medications (asthma, diarrhoea, pain relief, coughs and colds etc.) from the pharmacist whereas non-pharmacy town participants were not as consistent in support of one provider, χ^2 ^(6, *n *= 724) = 106.18, *p *< .001. Residents of three non-pharmacist towns were the main users of nursing posts. There was no significant difference between where respondents would go for advice on managing a specific illness, with 95% of both groups visiting the doctor. For advice on minor ailments there was a significant difference, χ^2 ^(4, *n *= 774) = 143.32, *p *< .001

Table 5 reveals that participants from pharmacy towns were much more likely to visit the pharmacist whereas participants from non-pharmacy towns were more likely to not seek any assistance.

**Table 5:** The health service that participants would seek advice from for managing a minor ailment (e.g. coughs, colds, sunburn) for pharmacy towns and non-pharmacy towns.

		Pharmacy Town	Non-pharmacy town	Total
Pharmacy	Count	224	85	309
		58.9%	21.6%	39.9%
Doctor	Count	45	51	96
		11.8%	12.9%	12.4%
Nursing post	Count	4	38	42
		1.1%	9.6%	5.4%
Local shop	Count	7	52	59
		1.8%	13.2%	7.6%
Stay and treat at home	Count	99	162	261
		26.1%	41.1%	33.7%
Other/not applicable	Count	1	6	7
		.3%	1.5%	.9%

**Table 6:** Opinions on provision of health services in local area for pharmacy town and non-pharmacy town participants

	Strongly Agree %	Agree %	Unsure %	Disagree %	Strongly Disagree%	Chi Square Test
Worry about suitability of prescriptions						χ2 (4, n = 729) = 11.76, *p *< .05
Pharmacy Town	6.6	25.3	14.5	35.1	18.5	
Non-pharmacy Town	5.4	25.4	18.0	40.9	10.3	
Enough advice about medications						χ2 (4, n = 736) = 21.15, *p *< .001
Pharmacy Town	18.7	58.0	11.1	10.6	1.6	
Non-pharmacy Town	10.0	55.7	13.7	15.4	5.1	
Too long a delay to access medications						χ2 (4, n = 737) = 49.54, *p *< .001
Pharmacy Town	2.9	10.0	5.8	54.6	26.8	
Non-pharmacy Town	9.0	19.4	7.0	54.2	10.4	
Travel too far to access medications						χ2 (4, n = 734) = 94.96, *p *< .001
Pharmacy Town	3.9	6.8	1.3	52.2	35.7	
Non-pharmacy Town	14.2	21.0	5.1	46.5	13.3	
Can get medications quickly in an emergency						χ2 (4, n = 736) = 36.46, *p *< .001
Pharmacy Town	21.8	49.3	13.9	11.0	3.9	
Non-pharmacy Town	10.4	44.2	14.4	23.4	7.6	
Privacy is important						χ2 (4, n = 739) = 6.68, *p *= .15
Pharmacy Town	33.9	51.2	5.2	8.1	1.6	
Non-pharmacy Town	28.1	51.1	6.5	12.9	1.4	
Good supply of medications at home						χ2 (4, n = 748) = 1.25, *p *= .87
Pharmacy Town	19.2	66.9	3.8	9.0	1.0	
Non-pharmacy Town	20.7	66.2	4.7	7.3	1.1	
Received good prevention advice						χ2 (4, n = 739) = 5.24, *p *= .26
Pharmacy Town	13.3	60.7	15.4	9.4	1.3	
Non-pharmacy Town	9.3	59.2	16.9	13.0	1.7	
Health services in town adequate for my needs						χ2 (4, n = 743) = 38.34, *p *< .001
Pharmacy Town	19.1	55.3	7.8	12.1	5.7	
Non-pharmacy Town	8.7	47.2	9.0	25.0	10.1	
Town needs more access to pharmacy services						χ2 (4, n = 735) = 187.27, *p *< .001
Pharmacy Town	6.0	11.8	12.0	52.9	17.3	
Non-pharmacy Town	30.6	31.4	14.4	21.5	2.0	

### Internet and mail order pharmacy use

Only 5.1% of pharmacy town residents and 5.8% of non-pharmacy town residents had purchased medicines over the internet. There was more support for a mail order service to obtain medicines, 16.6% of pharmacy town residents and 20.0% of non-pharmacy town residents. Only 23.3% of pharmacy town residents and 32.0% of non-pharmacy town residents were willing to use the internet or mail order to obtain medicines in the future.

### Opinions on health services

Table 6 presents the results on the opinion questions. For health services, 74.4% of pharmacy town participants either agreed or strongly agreed that health services were adequate compared to 55.9% of non-pharmacy town participants. For pharmacy services, 62.0% of non-pharmacy town participants thought their town needed more access to pharmacy services compared to around 17.8% of pharmacy town participants.

A large difference emerged on the whether participants thought there was too long a delay in obtaining medications, 30.3% of non-pharmacy town participants either strongly agreed or agreed that there was too long a delay compared to 12.9% of pharmacy town participants. Also 39.7% of non-pharmacy town participants thought they had to travel too far to have prescriptions filled compared to 10.7% of pharmacy town participants. Participants that obtained their medications from a pharmacist were more confident that they were receiving enough advice about medications, 75.9% agreed or strongly agreed, relative to those who obtained their medications from a doctor, 64.0% agreed or strongly agreed.

The last two items on the questionnaire enabled participants to provide their own comments as to the advantages of having a pharmacy and the disadvantages of not having a pharmacy. Basic medical advice, availability of prescriptions and pharmacy items, and no travel were common comments as to the advantages of having a pharmacy. Two of the pharmacy towns surveyed were serviced by the same pharmacist, splitting his time with mornings in one town and afternoons in the other town. Both these towns had patronage and satisfaction with health and pharmacy services figures comparable to the other pharmacy towns. There were some participants that thought that their town was not of sufficient size to support a pharmacy and were comfortable with the doctor dispensing arrangement. In addition, some participants expressed appreciation at being able to access medications from the doctor.

### Predicting satisfaction with services

After controlling all other variables, non-pharmacy town participants were twice as likely to be dissatisfied with the level of health services, *OR *= 2.02 (*CI *= 1.20 – 3.39) with the inclusion of towntype item making an independent contribution to health service satisfaction after inclusion of all the other variables, χ^2 ^(1) = 7.04, *p *< .01. Participants from non-pharmacy towns were eight times more likely to be dissatisfied with the level of pharmacy services in their town after controlling for the other variables, *OR *= 8.46 (*CI *= 4.99 – 14.35). The inclusion of towntype making an independent contribution to the prediction of pharmacy service satisfaction after inclusion of the health and demographic variables, χ^2 ^(1) = 71.23, *p *< .001.

#### Health Insurance Commission (HIC) data

**Figure 1:**
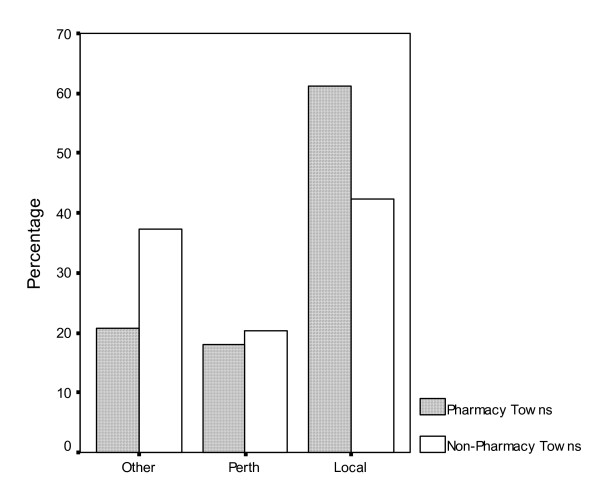
Dispensing location for pharmacy towns and non-pharmacy towns for all ages from 1^st ^January 2003 to 31^st ^December 2003.

The Health Insurance Commission produced data on dispensing rates for the 14 towns selected in this study. Due to privacy regulations within HIC, the data was aggregated into the two groups of pharmacy towns and non-pharmacy towns. The dispensing data refers to the number of scripts dispensed in three location categories for people residing within these towns (this was based on the resident's postcode as recorded on the script). The three location categories are in another town or state (Other), Perth metropolitan area (Perth) and home town (Local). Perth metropolitan area was classified according to the major statistical regions provided by the ABS and available on their web site [24]. These figures provided an opportunity to verify the health service patronage results of the questionnaire data. Overall there were 84,720 prescriptions dispensed for the pharmacy towns and 78,186 prescriptions dispensed for the non-pharmacy towns. Figure 1 displays the information for all age groups combined.

Similar to the questionnaire data more residents within pharmacy towns were having their prescriptions dispensed in their town relative to residents in non-pharmacy towns. The HIC data demonstrated the higher level of support for pharmacists relative to doctor dispensing services with 19% more prescriptions dispensed in the local town for pharmacy towns relative to non-pharmacy towns. While the relative difference between the two groups is consistent, the HIC data reveals a lower overall use of pharmacy services within their own town relative to the questionnaire data. The overall HIC figure of 61% of prescriptions dispensed in the local town for pharmacy towns was lower than the questionnaire data. Some of this discrepancy can be attributed to the high use of pharmacy services in a town not included in this study which bordered two of the selected pharmacy towns. Those on the border of either included shire may have considered the pharmacy from this other town as their 'local' pharmacy in the questionnaire.

## Discussion

The results of the study have been discussed according to the primary objectives of the study.

### 1. To determine the pattern of patronage of pharmacy and doctor services in small rural towns

Both the questionnaire and HIC data indicated a strong preference for pharmacy services over doctor dispensing services. Where a town can support a pharmacy it would seem there is overwhelming support for this model over a doctor dispensing model. There was some support expressed in the comments section for the dispensing doctor model which is consistent with the aim of improving access to pharmaceutical care in rural areas that cannot support a pharmacy practice [13]. However, as the towns selected were of equal size it would seem that some doctor dispensing licences exist where a pharmacist could be supported, which denies the town's population access to this service [25-27].

Anecdotally the authors are aware that the pharmacists consider the percentage of older people residing within a town when making decisions about establishing a pharmacy service. However, while there were a higher percentage of older people living in pharmacy towns there was no difference in the use of medications. Greater clarification of the population statistics required to support a pharmacy is required so that doctor dispensing licences are only granted where a pharmacy would not be viable.

In the introduction, telepharmacy and mobile pharmacy were discussed as two possible models of pharmacy care that could be used in rural areas. There was low support for both mail and internet options with only a quarter to a third of participants willing to use such services in the future. While this does suggest a sizeable market for such programs it would not be sufficient as a model to meet the pharmacy needs of a rural town. There could be more promise in a mobile pharmacy model. In this model, a pharmacist travels with supplies and rotates among several small towns that do not have a pharmacy [10,11]. It has been contended that this may assist in reducing travel time for rural residents or, alternatively, reduce the waiting time on having prescriptions filled [12]. The two towns receiving part-time pharmacy services had the same results for access and satisfaction as the other pharmacy towns. Further research could investigate whether a mobile service that provided daily access, even if for a smaller period of time, could meet the financial needs of the pharmacist and health needs of the community.

### 2. To determine access and availability of prescription and non-prescription medicines in rural communities

The speed at which these medicines were obtained would seem to have differed markedly between the town types. One of the most distinctive differences between the town types was travel. Participants from non-pharmacy towns were much more likely to indicate that they had to travel too far or that there was a delay in accessing medication. The questionnaire results and participants' comments corroborate previous research that has identified travel and fuel costs as barriers to accessing pharmacy services [5,28,29].

### 3. To provide data on how minor ailments are managed in towns with and without pharmacies

Pharmacy town participants were much more likely to seek advice from a pharmacist for managing a minor ailment whereas non-pharmacy town participants were much more likely to treat the problem at home and not seek advice. This corresponds with previous research that has found that the local pharmacy is often the first place people go to seek assistance with minor ailments or basic information on health issues and products [30,31]. For purchasing items related to managing minor ailments, hayfever, coughs and colds, constipation, diarrhoea, the majority of pharmacy town participants used the pharmacy whereas around one third of non-pharmacy town participants would purchase the items from the local shop. People may be able to manage their minor ailments without health professional advice. However, this tendency to not seek assistance may be problematic if the problem was more serious. Distance and travel has been mentioned as a possible reason why rural people are much less likely than urban people to seek help about medical problems, particularly when such problems are perceived as trivial or intermediate [11,32,33].

## Conclusions and recommendations

There are some limitations to this study which need to be acknowledged when making recommendations as to health service delivery and further research recommendations. While a range of towns were selected in the study there still could have been particular personalities/professional competencies of the health service providers that may have biased the results or particular features with the populations studied which may not translate to other communities. As mentioned, information was not available on doctor dispensing licences and town selection was made according to matching single pharmacy towns as closely as possible to the known towns where a dispensing doctor was operating. This may limit whether the results can be translated to other rural regions. However, when choosing towns consideration was given to ensuring they were from a range of geographical locations. Further the large and consistent difference between pharmacy towns and non-pharmacy towns would suggest that the finding is a reliable indicator of difference in pharmaceutical service access between pharmacy towns and non-pharmacy towns.

While the close matching of the two town groups provides strength as a comparative study there were limitations in ascertaining a complete picture of rural health service usage. The response rate was low to moderate and perhaps could have been improved by offering incentives or follow up reminders beyond the second mail out [34]. Another factor could have been the length of the questionnaire although this was tested on a focus group of health consumers prior to the pilot study to ensure its ease of completion. Another limitation was that the majority of respondents were female yet the ABS data revealed that the towns had higher percentages of males than females. While respondents were asked to respond on behalf of the family, female respondents answering on behalf of their partners might not accurately reflect male use of health services. Also it is possible that a member of the family in better health completed the questionnaire and did not accurately reflect the views of someone in the household with chronic health conditions. However, there were no differences in health status or medication use between the two town groups that if present would have confounded the results.

The main finding of the study was that non-pharmacy town participants were more likely to be dissatisfied with the level of health and pharmacy service access they were receiving. The most salient features of this dissatisfaction were having to travel to access medications and not being able to immediately access medications from the doctor's dispensary. More non-pharmacy town participants thought they were receiving inadequate advice on medicines. Thus there is a clear preference for pharmacy services where they can be adequately supported by the community. As the towns selected in this study were of similar size it is difficult to ascertain the criteria used for doctor dispensing licences to be granted. Further research is required on the economic sustainability of pharmacies in small rural communities and a clearer benchmark provided so that doctor dispensing licences are appropriately granted.

## Competing interests

The author(s) declare that they have no competing interests.

## Authors' contributions

VBS and SDB jointly conceived of the study, wrote the questionnaire, and helped draft the manuscript. AWJ coordinated the study, performed the statistical analysis and drafted the manuscript.
